# Troponin I is an independent predictor of cardiovascular events and mortality in haemodialysis patients

**DOI:** 10.1093/ckj/sfaf047

**Published:** 2025-02-11

**Authors:** Maria Tydén, Magnus E Westerlund, Kevin Duarte, Niclas Eriksson, Nicolas Girerd, Bernhard K Krämer, Winfried März, Patrick Rossignol, Hubert Scharnagl, Inga Soveri, Maria K Svensson, Faiez Zannad, Bengt Fellström

**Affiliations:** Department of Medical Sciences, Renal Medicine, Uppsala University, Uppsala, Sweden; Department of Medical Sciences, Renal Medicine, Uppsala University, Uppsala, Sweden; Université de Lorraine, Centre d'Investigations Cliniques Plurithématique 1433 and Inserm U1116, CHRU Nancy, FCRIN INI-CRCT (Cardiovascular and Renal Clinical Trialists), Nancy, France; Uppsala Clinical Research Center (UCR), Uppsala University, Uppsala, Sweden; Université de Lorraine, Centre d'Investigations Cliniques Plurithématique 1433 and Inserm U1116, CHRU Nancy, FCRIN INI-CRCT (Cardiovascular and Renal Clinical Trialists), Nancy, France; Vth Department of Medicine, University Medical Centre and Medical Faculty Mannheim, Heidelberg University Mannheim, Germany; Vth Department of Medicine, University Medical Centre and Medical Faculty Mannheim, Heidelberg University Mannheim, Germany; Clinical Institute of Medical and Chemical Laboratory Diagnostics, Medical University of Graz, Austria; SYNLAB Academy, SYNLAB Holding Deutschland GmbH, Augsburg and Mannheim, Germany; Université de Lorraine, Centre d'Investigations Cliniques Plurithématique 1433 and Inserm U1116, CHRU Nancy, FCRIN INI-CRCT (Cardiovascular and Renal Clinical Trialists), Nancy, France; Department of Medicine and Nephrology-Hemodialysis, Princess Grace Hospital, and Monaco Private Hemodialysis Centre, Monaco, Monaco; Clinical Institute of Medical and Chemical Laboratory Diagnostics, Medical University of Graz, Austria; Department of Medical Sciences, Renal Medicine, Uppsala University, Uppsala, Sweden; Department of Medical Sciences, Renal Medicine, Uppsala University, Uppsala, Sweden; Uppsala Clinical Research Center (UCR), Uppsala University, Uppsala, Sweden; Université de Lorraine, Centre d'Investigations Cliniques Plurithématique 1433 and Inserm U1116, CHRU Nancy, FCRIN INI-CRCT (Cardiovascular and Renal Clinical Trialists), Nancy, France; Department of Medical Sciences, Renal Medicine, Uppsala University, Uppsala, Sweden

**Keywords:** cardiovascular disease, haemodialysis, mortality, troponin I

## Abstract

**Background:**

Patients with end-stage kidney disease (ESKD) undergoing haemodialysis (HD) have a high risk of cardiovascular (CV) events. This study evaluated troponin I (hs-cTnI) as a predictor of major adverse cardiac events (MACEs), CV death and all-cause death.

**Methods:**

The AURORA trial, a multicentre, randomized, double-blind trial involved 2776 HD patients comparing rosuvastatin with placebo. No significant effect was found on the composite primary endpoint of CV death, non-fatal myocardial infarction or non-fatal stroke. In this *post hoc* analysis, we analysed the association between baseline hs-cTnI and outcomes using Cox regression analyses. We adjusted for multiple background factors and available biomarkers. Hs-cTnI was log_2_-transformed and modelled using a four-knot restricted cubic spline. Variables were ordered by their importance in the models using χ^2^ value minus degrees of freedom.

**Results:**

Baseline median hs-cTnI was 17.3 pg/mL. During follow-up, 734 MACEs, 598 CV deaths, and 1094 total deaths occurred. Patients in the upper quartile of hs-cTnI (>32.6 pg/mL) had significantly higher risk of MACEs [hazard ratio (HR) 1.92; 95% confidence interval (CI) 1.57–2.35], CV death (HR 2.12; 95% CI 1.69–2.66), all-cause death (HR 1.84; 95% CI 1.55–2.17) and non-CV death (HR 1.59; 95% CI 1.23–2.05) after full adjustment compared with those in the lowest quartile (<10.1 pg/mL). Hs-cTnI was identified as the strongest predictor for MACEs, CV death, and all-cause death, but not for non-CV death.

**Conclusions:**

Baseline hs-cTnI is a strong and independent predictor for MACEs and death in patients with ESKD undergoing haemodialysis.

KEY LEARNING POINTS **What was known:**

- Major adverse cardiovascular events (MACEs) are common in patients with end-stage kidney disease (ESKD) and haemodialysis (HD) treatment and constitute the main cause of death in this patient group. The pathophysiology is complex, multifactorial, and incompletely understood.

**This study adds:**

- In this retrospective analysis of 2342 patients with ESKD and HD treatment, the role of high-sensitivity troponin I (hs-cTnI) was studied. Hs-cTnI was found to be an independent risk marker for cardiovascular disease and death, outranking other measured traditional and emerging risk markers.

**Potential impact:**

- The mechanisms for the association between cardiac troponins and risk increase remain to be elucidated. Cardiovascular risk assessment in patients with ESKD and HD treatment should include cardiac troponins.

## INTRODUCTION

End-stage kidney disease (ESKD) is associated with an increased risk of cardiovascular (CV) mortality and morbidity, with haemodialysis (HD) patients facing a 10- to 20-fold higher risk than those with normal kidney function [1]. The reasons for the increased risk are incompletely understood. Traditional CV risk markers, such as hypertension, diabetes mellitus, and left ventricular hypertrophy (LVH), are prevalent in HD patients [2, 3]. In addition, factors related to uraemia, including anaemia, fluid retention, hyperphosphataemia, secondary hyperparathyroidism, endothelial dysfunction, oxidative stress, and chronic inflammation, may contribute [4–7]. Trials targeting modifiable risk markers, such as anaemia and serum lipid levels, have yielded neutral results in the HD population [8–10]. Cardiac troponin I (cTnI) and T (cTnT) are released by myocytes following cardiac injury. They hence have high sensitivity for cardiac injury, being the preferred biomarkers in diagnosing acute myocardial infarction (MI) [11, 12]. However, they also rise in non-necrotic conditions, such as strenuous exercise or brief ischaemia [13]. High-sensitivity assays of troponin I and T (hs-cTnI and hs-cTnT) have emerged during the last decade and are recommended for detecting myocardial injury in the setting of chest pain [14, 15]. Troponins are prognostic markers in acute and chronic heart failure [16, 17]. Studies also link hs-cTn levels in asymptomatic patients to increased CVD risk [18–20]. A systematic review of 98 studies found that elevated cTn is strongly associated with an increased risk of major adverse cardiovascular events (MACEs), including CV death, in patients with chronic kidney disease (CKD) [21]. Limited data on hs-cTnI and hs-cTnT in ESKD patients on HD suggest that elevated hs-cTn levels are associated with an increased risk of adverse outcomes [22, 23]. This study aimed to investigate hs-cTnI as a predictor of CV events and death alongside other established and emerging CV risk markers using data from AURORA (A Study to Evaluate the Use of Rosuvastatin in Subjects on Regular Haemodialysis: An Assessment of Survival and Cardiovascular Events trial) [8].

## MATERIALS AND METHODS

### Study population

The AURORA study was an international, multicentre, randomized (1:1), double-blind clinical trial, in which 2776 patients aged 50–80 with ESKD receiving maintenance HD for at least 3 months were randomly assigned to rosuvastatin 10 mg daily or matched placebo and followed up for a median of 3.8 years [8]. Details of the AURORA study database have been described (NCT04042350) [24]. This *post hoc* analysis was performed using the subset of the AURORA study database with available baseline hs-cTnI measurements. The study was conducted in accordance with the Declaration of Helsinki, the International Conference of Harmonization/Good Clinical Practice guidelines, and local regulatory requirements. All patients provided written informed consent, and the ethics committee at each centre approved the trial, including *post hoc* analyses.

### Outcomes

The composite primary endpoint MACE was defined as non-fatal MI or non-fatal stroke or death from CV causes. Primary endpoints were reviewed and adjudicated by a clinical endpoint committee blind to treatment allocation. Secondary endpoints included death from all causes and death from CV and non-CV causes. Additionally, the individual components of MACEs, specifically MI and stroke, were analysed separately. Treatment with rosuvastatin was not associated with a reduction in the composite primary endpoint of MACE.

### Risk factors and risk markers

We considered risk factors or markers of different categories: (i) traditional risk factors (e.g. age, previous CV events, hypertension, dyslipidaemia, diabetes mellitus, and smoking) [25]; (ii) disease-specific risk markers associated with CKD (e.g. uraemic burden, time on dialysis treatment, anaemia, and altered calcium phosphate metabolism) [3, 7, 26]; (iii) emerging risk markers, including markers of cardiac disease, such as hs-cTnI, B-type natriuretic peptide (BNP), and markers of anaemia or systemic inflammation, including ferritin, transferrin, and galectin 3 [4–6].

### Biochemical methods

Blood samples were drawn before the start of the HD session in the middle of the dialysis week, and mostly analysed during the time of the original study [27]. BNP and hs-cTnI analyses were performed using a chemiluminescent microparticle immunoassay (CMIA) (Abbott Architect i2000 Analyzer, Abbott Diagnostics, Chicago, IL, USA). The coefficients of variation for BNP were 2.1%, 2.8%, and 0.8% (within run), and 5.2%, 5.8%, and 6.1% (between runs) at concentrations of 86, 442, and 3330 pg/mL, respectively. Coefficients of variation for hs-cTnI were 2.1%, 2.6%, and 2.0% (within run) and 4.4%, 5.7%, and 5.8% (between runs) at concentrations of 21, 195, and 15 310 pg/mL, respectively.

### Statistical methods

Descriptive statistics used percentages for categorical variables and median (interquartile range) for continuous variables. Baseline characteristics were presented within the quartile groups and combined. Confidence intervals for incidence rates were calculated using a γ distribution. Analyses were performed using univariate and multivariate Cox regression models for all endpoints of the study. For descriptive purposes, hs-cTnI was categorized into quartile groups. A log_2_ transformation was applied to all biomarkers and to years on HD (estimates are presented on the original scale). To allow for non-linear associations, all continuous variables were modelled using four-knot restricted cubic splines with knots at the 5th, 35th, 65th, and 95th percentiles of each variable's distribution. Hazard ratios for hs-cTnI are presented for the two versus the lowest quartile of the distribution and the full association with each outcome are presented graphically as model-predicted 3-year risks of event. Because associations between hs-cTnI and outcomes were assessed using Cox regression models including hs-cTnI modelled using a restricted cubic spline, it was not possible to give one single hazard ratio to summarize the association. Instead, we graphically describe the association. Cumulative event rates were examined using the Kaplan–Meier method and were plotted per quartile group of hs-cTnI.

Both unadjusted and multiple models, adjusting for relevant potential confounders, were fitted. Adjusted models included age and sex (model 2) and, in addition adjustment for origin, current smoking, diabetes mellitus, history of coronary heart disease (CHD), atrial fibrillation, systolic blood pressure (SBP), diastolic blood pressure (DBP), heart rate, height, weight, years on HD treatment, treatment with rosuvastatin, haemoglobin, LDL-C, HDL-C , triglycerides, high-sensitivity C-reactive protein (hs-CRP), albumin, phosphate, erythropoietin treatment, use of angiotensin-converting enzyme inhibitor, anticoagulation treatment, sevelamer use, iron supplements, ferritin, transferrin, galectin 3, BNP, C-terminal pro-peptide of collagen type 1 (CICP), and stem cell factor (SCF) (model 3). Missing data, e.g. plasma samples not available (up to 14.4% for CICP and SCF) were multiply imputed using the R-package MICE and 10 imputed sets. Presented Cox regression results arise from multiple imputations unless otherwise stated. The *C*-statistic (Harrell's *C*) was used to quantify each model's ability to discriminate between occurrence or absence of an event. Fraction of new information (FNI) was calculated as 1 − (model1 likelihood ratio χ^2^/model2 likelihood ratio χ^2^), where model1 is a model without the marker of interest and model2 is the full model. All analyses were performed using R version 4.1.2 (R Foundation for Statistical Computing, Vienna, Austria) with the rms and Hmisc packages.

## RESULTS

A total of 2342 patients with a baseline measurement of hs-cTnI were included in this study (Table 1). Median hs-cTnI was 17.3 (10.1–32.6) pg/mL. The baseline characteristics of the population stratified by hs-cTnI quartiles are presented in Supplementary Table 1. The subset of patients for whom hs-cTnI was available had clinical characteristics similar to the one in which hs-cTnI was missing (Supplementary Table 2). An overview of primary and secondary endpoints, number of events, median and maximal follow-up, and the incidence rates, measured as events per 100 patient years, is shown in Table 2. The partial *R*^2^ describes to what extent the variance of hs-cTnI was affected by the other measured variables. As shown in Supplementary Fig. 1, BNP, the factor affecting hs-cTnI to the greatest extent, contributed 7% of the variance of hs-cTnI; age contributed <2%.

**Table 1:** Baseline characteristics.

	*N*	Median [Q1–Q3]
hs-cTnI (pg/mL)	2342	17.3 [10.1–32.6]
Age	2342	64 [56–72]
SBP (mmHg)	2340	138 [120–150]
DBP (mmHg)	2339	78 [68–83]
Pulse pressure	2339	60 [50–72]
Heart rate	2258	76 [68–84]
Height (cm)	2320	167 [160–173]
Weight (kg)	2333	69 [59–79]
Years on haemodialysis treatment	2341	2.36 [1.03–4.46]
Haemoglobin (g/L)	2302	117 [107–127]
Haematocrit (ratio)	2257	0.35 [0.32–0.38]
Cholesterol (mmol/L)	2333	4.40 [3.78–5.18]
LDL-C (mmol/L)	2333	2.50 [1.95–3.11]
HDL-C (mmol/L)	2333	1.09 [0.91–1.35]
Triglycerides at baseline (mmol/L)	2333	1.46 [1.05–2.10]
hs-CRP (mg/L)	2333	5.13 [2.1–14.3]
Albumin (g/L)	2339	40 [37–42]
Phosphate (mmol/L)	2339	1.74 [1.42–2.10]
Ferritin (mg/dL)	2230	444 [233–769]
Transferrin (ng/mL)	2230	174 [150–198]
Galectin 3 (ng/mL)	2342	65.8 [51.7–84.4]
BNP (pg/mL)	2342	115 [39–317]
CICP (ng/mL)	2004	153 [117–214]
SCF (ng/mL)	2004	3.80 [3.08–4.61]
Ethnicity, *N* (%)		
Caucasian	2342	1985 (85%)
Black		78 (3%)
Asian		128 (5%)
Hispanic		93 (4%)
Other		58 (2%)
Sex female, *N* (%)	2342	888 (38%)
Current smoker: yes, *N* (%)	2342	362 (15%)
Diabetes: yes, *N* (%)	2342	604 (26%)
CHD history: yes, *N* (%)	2342	1191 (51%)
Atrial fibrillation: yes, *N* (%)	2342	258 (11%)
Treatment with rosuvastatin 10 mg, *N* (%)	2342	1178 (50%)
EPO treatment: yes, *N* (%)	2342	2067 (88%)
ACE use: yes, *N* (%)	2339	852 (36%)
Anticoagulation treatment: yes, *N* (%)	2342	231 (10%)
Sevelamer use: yes, *N* (%)	2339	447 (19%)
Iron supplements: yes, *N* (%)	2342	1068 (46%)

Values are median (Q1–Q3) for continuous variables and frequency (percentage) for categorical variables.

*N* is the number of data available for each variable.

ACE, ACE inhibitors; HDL, high-density lipoprotein; LDL low-density lipoprotein; EPO, erythropoietin-stimulating agents.

**Table 2:** Descriptive for the outcomes where hs-cTnI was measured.

Outcome	Number	Number of events	Median follow-up (years)	Maximal follow-up (years)	Patient years	Incidence rate Median and quartile range
MACE	2342	734	3.85	5.52	7557.71	9.71 [9.02–10.44]
CV death	2342	598	3.89	5.56	7861.72	7.61 [7.01–8.24]
Death (overall)	2342	1094	3.9	5.56	7887.62	13.87 [13.06–14.72]
Non-CV death	2342	496	3.9	5.56	7887.62	6.29 [5.75–6.87]

### MACE

**Figure 1:**
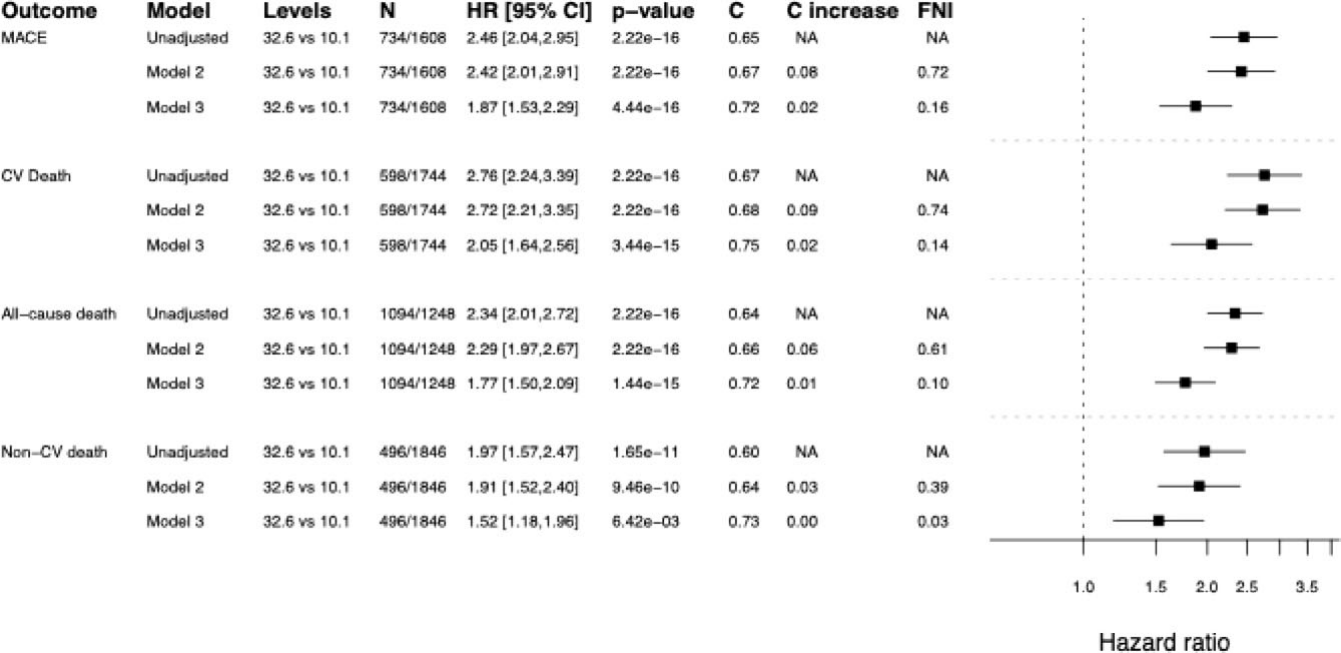
Forest plot for the effect of hs-cTnI in different models and for different outcomes. *N* shows the numbers of events/number of non-events. For multiple models the increase in *C*-statistic and fraction of new information (FNI) by adding hs-TnI is presented. Model 2 includes adjustment for age and sex. Model 3 includes adjustment for age, origin, sex, current smoker and diabetic status, CHD history, atrial fibrillation, SBP (mmHg), DBP (mmHg), heart rate, height (cm), weight (kg), years on haemodialysis treatment, treatment with rosuvastatin, haemoglobin (g/L), cholesterol (mmol/L), LDL-C (mmol/L), HDL-C (mmol/L), triglycerides at baseline (mmol/L), hs-CRP (mg/L), creatinine (mg/dL), albumin (g/L), phosphate (mmol/L), EPO treatment, ACE use, anticoagulation treatment, sevelamer use, iron supplements, ferritin (mg/dL), transferrin (ng/mL), galectin 3 (ng/mL), BNP (pg/mL), CICP (ng/mL), and SCF (ng/mL). S, serum;.

During the median follow-up time of 3.85 years, 734 patients (31%) experienced a MACE (Table 2). As presented in Supplementary Table 3, the endpoint is predominantly driven by CV death (*n* = 598), followed by non-fatal MI (*n* = 177) and non-fatal stroke (*n* = 89). In univariate analyses, hs-cTnI was associated with the risk of MACE [highest versus lowest quartile; hazard ratio (HR) 2.45; 95% confidence interval (CI) 2.04–2.95; *P* < .001] (Fig. 1)

**Figure 2:**
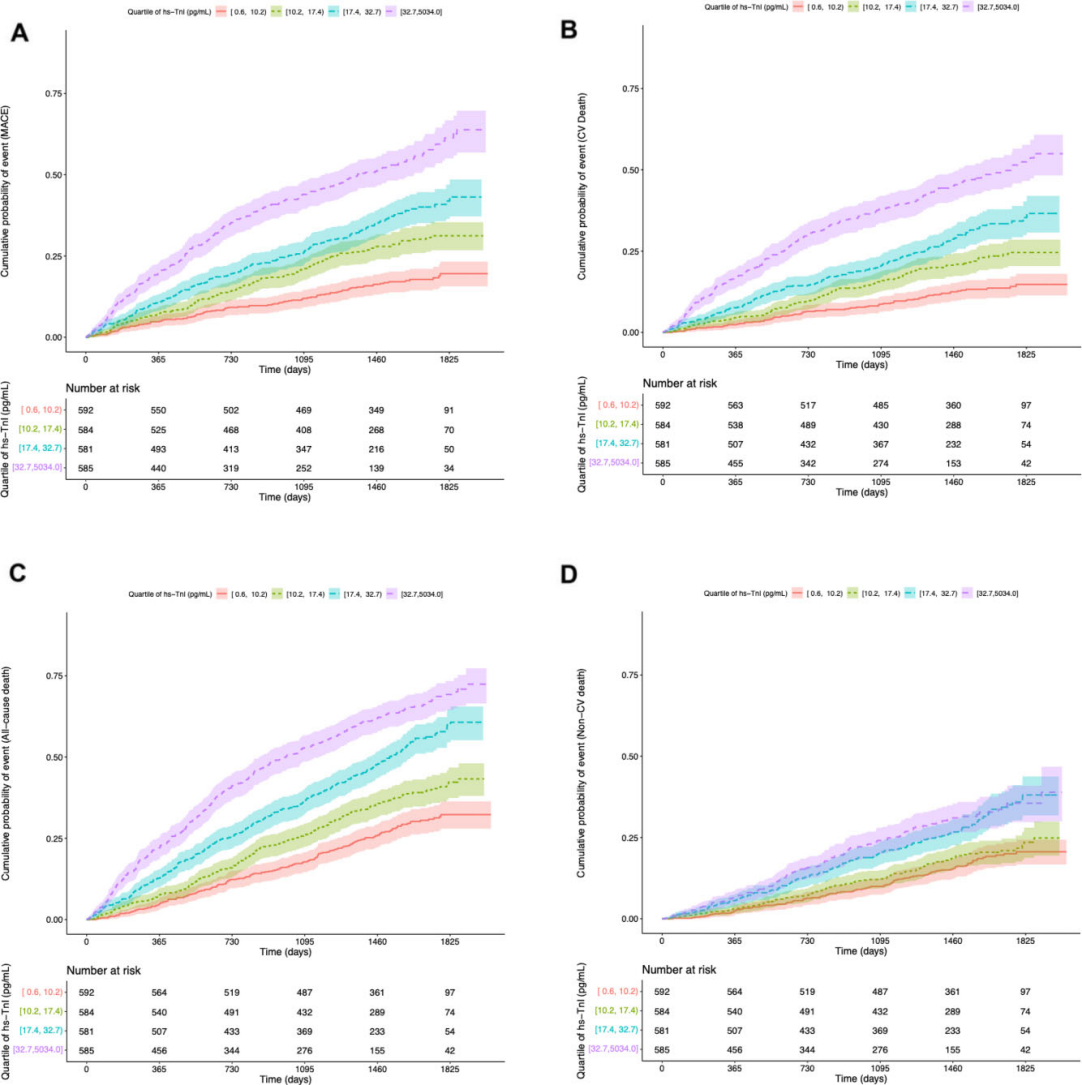
Kaplan–Meier estimate of the cumulative event rate of MACE (A), CV death (B), all-cause death (C), and non-CV death (D) by quartile groups of hs-cTnI.

**Figure 3:**
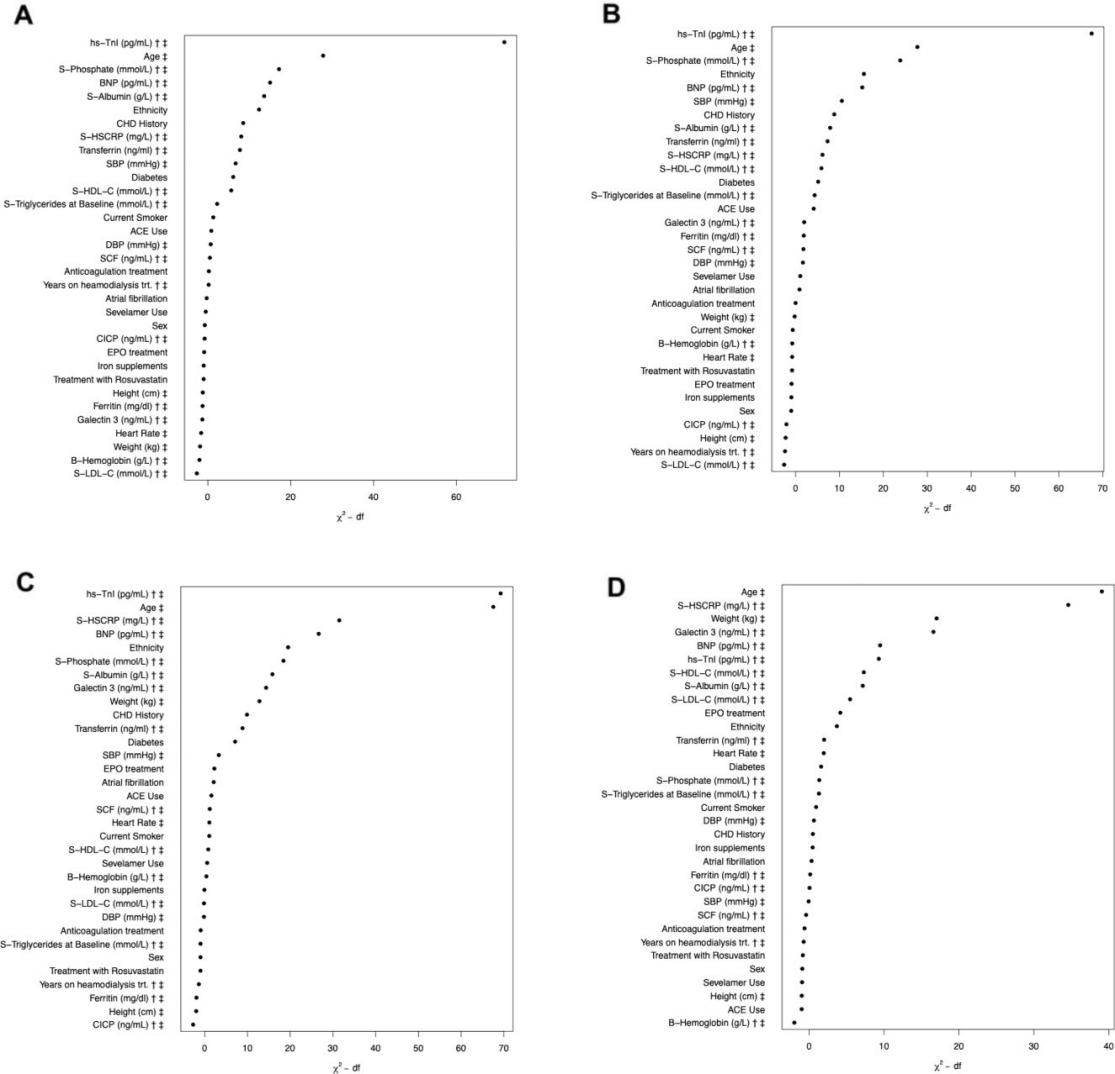
(A–D) Variables ordered by variable importance estimated by χ^2^ value: degrees of freedom (df) for a model including all predictors for the outcome MACE, CV death, all-cause death, and non-CV death. Symbols after marker name: †, log_2_-transformed; ‡, modelled using a restricted cubic spline. ACE use means use of ACE inhibitors; S, serum; HDL, high-density lipoprotein; LDL low-density lipoprotein; EPO, erythropoietin stimulating agents.

The unadjusted association between hs-cTnI and 3-year risk of MACE is presented in Supplementary Fig. 2A. The cumulative risk of MACE by hs-cTnI quartiles is presented as a Kaplan–Meier plot (Fig. 2A). In a model adjusted for age and sex, hs-cTnI remained associated with MACE risk (HR 2.42; 95% CI 2.01–2.91; *P* < .001). In a fully adjusted model, hs-cTnI remained associated with MACE risk (HR 1.92; 95% CI 1.57–2.35; *P* < .001) (Fig. 1). Figure 3A depicts the importance of hs-cTnI in the multiple Cox regression model for MACE including all prespecified covariates. When analysing the individual components of MACE (not only CV death but also MI and stroke), hs-cTnI was the most important variable for MI and the second most important variable for stroke, in multiple Cox regression models (Supplementary Fig. 3A, B).

### CV death

There were 598 cases (26%) of CV death during the follow-up time (Table 2). In univariate analyses, hs-cTnI was associated with CV death (highest versus lowest quartile; HR 2.76; 95% CI 2.24–3.39; *P* < .001) (Fig. 1). The unadjusted predicted 3-year risk of CV death versus hs-cTnI is presented in Supplementary Fig. 2b. The cumulative risk of CV death by hs-cTnI quartiles is depicted as a Kaplan–Meier plot (Fig. 2B). In a model adjusted for age and sex, hs-cTnI remained associated with the risk of CV death (HR 2.72; 95% CI 2.21–3.35; *P* < .001). In a fully adjusted model, hs-cTnI remained associated with CV death (HR 2.12; 95% CI 1.69–2.65; *P* < .001) (Fig. 1). Figure 3B depicts the importance of hs-cTnI in the multiple Cox regression model for CV death including all prespecified covariates.

### All-cause death

A total of 1094 patients died (47%) (Table 2). In univariate analyses, hs-cTnI was associated with all-cause death (highest versus lowest quartile; HR 2.34; 95% CI 2.01–2.72; *P* < .001) (Fig. 1). The predicted 3-year risk of all-cause death versus hs-cTnI is presented in Supplementary Fig. 2c. The cumulative risk of all-cause death by hs-cTnI quartiles is depicted as a Kaplan-Meier plot (Fig. 2c). In a model adjusted for age and sex, hs-cTnI remained associated with all-cause death (HR 2.29; 95% CI 1.97–2.67; *P* < .001). In a fully adjusted model, hs-cTnI remained associated with all-cause death (HR 1.84; 95% CI 1.55–2.17; *P* < .001) (Fig. 1). Figure 3C depicts the importance of hs-cTnI in the multiple Cox regression model for all-cause death including all prespecified covariates.

### Non-CV death

A total of 496 patients (21%) died due to non-CV causes (Table 2). In univariate analyses, hs-cTnI was associated with non-CV death (highest versus lowest quartile; HR 1.97; 95% CI 1.57–2.47; *P* < .001) (Fig. 1). The predicted 3-year risk of non-CV death versus hs-cTnI is presented in Supplementary Fig. 2d. The cumulative risk of non-CV death in hs-cTnI quartiles is depicted as a Kaplan–Meier plot in Fig. 2D. In a model adjusted for age and sex, hs-cTnI remained associated with non-CV death (HR 1.91; 95% CI 1.52–2.34; *P* < .001). In a fully adjusted model, hs-cTnI remained associated with non-CV death (HR 1.59; 95% CI 1.23–2.05; *P* = .00153) (Fig. 1). Figure 3D depicts the importance of hs-cTnI in the multiple Cox regression model for non-CV death including all prespecified covariates. Hs-cTnI was outranked by age, hs-CRP, galectin 3, and weight.

### Interaction analyses

A post review analysis of the interaction between hs-cTnI and the subgroups atrial fibrillation (AF), CHD, rosuvastatin treatment, and BNP levels, in relation to the outcomes of MACE, CV death, all-cause death, and non-CV death, was performed (statistical analysis is presented in the supplementary material). Across all models hs-cTnI consistently shows a strong and significant association with increased risk of MACE, CV death, all-cause death, and non-CV death. For AF some *P*-values for interaction (*P*_int_) suggest potential effect modification for MACE and CV death but lack consistent statistical significance for all models and outcomes (*P*_int_ ranges from 0.025 to 0.451 for different outcomes and models). There was no significant interaction between hs-cTnI and CHD history, or hs-cTnI and rosuvastatin treatment, for all outcomes, and *P*_int_ values for the hs-cTnI and BNP interaction indicate a weak or no significant modification of the relationship between hs-cTnI and outcomes (Supplementary Fig. 4A–D).

## DISCUSSION

Using the AURORA trial database, we demonstrate that baseline hs-cTnI is an independent and strong predictor of MACE, CV death and all-cause death in patients with ESKD on HD. In our model hs-cTnI outranked age, phosphate, diabetes mellitus, BNP, hs-CRP, and a history of previous coronary events.

Remarkably, for all-cause death, hs-cTnI outranked age, a well-established risk factor in the general population and in disease-specific populations [28]. Age was the strongest risk marker for non-CV death. Besides hs-cTnI and age, elevated BNP, phosphate, and hs-CRP were also associated with MACE and CV mortality and all-cause mortality. Previous CHD, diabetes mellitus, and low albumin also indicated higher risk. For non-CV death, age, hs-CRP, galectin 3 and weight outperformed hs-cTnI. The predictive power of hs-cTnI was not meaningfully altered by subgroup factors in this study. This finding reinforces hs-cTnI's role in guiding risk stratification and management in patients at risk of CV events, without the need for subgroup-specific adjustments.

The mechanisms underlying the association between elevated cTn and increased mortality in patients with ESKD on HD remain speculative. Decreased renal clearance of cTn may contribute to increased concentrations, but this is unlikely to be the whole explanation [29]. Increased cTn may mark the presence of uraemic cardiomyopathy, a phenotype of cardiac disease prevalent in patients with CKD and best characterized by diastolic dysfunction, LVH, and myocardial fibrosis [30]. Uraemic cardiomyopathy is believed to have a complex multifactorial pathophysiology, including haemodynamic overload, anaemia, chronic kidney disease–bone mineral disorder, and accumulation of uraemic toxins [30–32]. cTn leakage is reported in the absence of MI and observed in conditions like heart failure, sepsis, myocarditis, and strenuous exercise [13, 33–35]. Subclinical cardiac ischaemia could also contribute to elevated cTn. The mechanism of myocardial injury in these states differs from that of MI, i.e. ischaemia-induced necrosis of myocytes. In a murine model, elevated cTnI was the result of increased preload, dissociated from myocardial stunning, and it has been speculated that preload-induced cTnI leakage could impair myocardial function [36]. Uçar *et al*. reported that increased levels of hs-cTnT in patients with newly diagnosed hypertension were associated with LVH and parameters of left ventricular remodelling [37]. Our study demonstrates that hs-cTnI has the greatest impact when predicting the risk of MACE, CV mortality, and all-cause mortality in patients with HD, surpassing established risk markers such as BNP, age, previous CV events, hypertension, dyslipidaemia, diabetes mellitus, and smoking. The finding that elevated cTn plays a significant role as a risk marker for MACE, CV mortality, and all-cause mortality in patients with CKD with or without dialysis is in agreement with previous studies [22, 38]. A meta-analysis concluded that elevated cTnT identified a subgroup of patients with ESKD that had a high risk of cardiac death and poor survival rate in the absence of symptoms suggesting myocardial ischaemia [39]. Although studies on hs-cTn in HD patients are limited, they consistently report that elevated hs-cTn is associated with poor prognosis [22]. When evaluating methods and interventions to prevent CVD in HD, monitoring cTn could serve as a risk enrichment marker, selecting patients at high CV risk and perhaps the TnI reference should be revised and adapted to HD patients.

Timing of TnI blood sampling is important due to conflicting results as to whether HD impacts on cTn, with observed decreases, increases, or no changes [40–42]. cTn increases during HD are associated with worse cardiac outcomes [41]. These previous findings warrant further investigations. Studies designed to establish standardized sampling protocols for cTnI could be useful. Alongside hs-cTnI and age, elevated BNP was an independent risk marker. Previous studies show that elevated proBNP and NT-proBNP are sensitive and specific predictors of MACE in HD patients [43–45]. Our finding that BNP is the most significant covariate explaining the variance in hs-cTnI levels suggests that hs-cTnI elevations reflect heart failure-associated myocardial injury. However, the modest proportion of variance explained by BNP underscores that hs-cTnI is impacted by multiple injury mechanisms, involving factors such as inflammation and ischaemia. LVH, common in the HD population, can cause symptoms of cardiac failure and cTn release, as seen in hypertensive patients [37, 46]. Inflammation, marked by elevated hs-CRP, is common in CKD and more so in HD [47, 48]. Inflammation contributes to the MIA syndrome (malnutrition, inflammation, atherosclerosis) linked to high mortality [49]. Targeting inflammation with modern biological anti-inflammatory agents, like interleukin blockers, could be a future treatment also in HD patients.

To the best of our knowledge, this is the first study to rank and combine emerging risk markers (hs-CRP, hs-cTnI, BNP) against traditional and disease-specific ones in HD. In addition to observational studies, randomized control trials could be designed to evaluate the impact of interventions based on hs-cTnI levels. Patients with elevated hs-cTnI levels could be stratified into different treatment arms to determine whether targeted therapies such as intensified CV monitoring or anti-inflammatory treatment could improve outcomes. Incorporating hs-cTnI into routine clinical assessment would require establishing standardized thresholds for risk stratification, as well as educating healthcare providers on the interpretation of hs-cTnI levels in the context of CKD and HD. To integrate hs-cTnI into clinical practice, the development of a risk prediction model that incorporates hs-cTnI alongside other established risk markers—such as BNP, age, and comorbidities—would be instrumental. This present work is the first step in developing a risk prediction tool to estimate the outcome for individual patients of an HD cohort.

A major strength of the AURORA trial is its large, well-characterized cohort of HD patients from 25 countries. Events were adjudicated by an independent data and safety monitoring board using prespecified criteria. To appreciate the findings, limitations need to be addressed; the study is a *post hoc* analysis and the associations observed do not establish causality. Patient recruitment occurred 20 years back (year 2003–04), and some of the treatment guidelines have evolved since then. Residual confounding from measurement errors, unmeasured factors, and the lack of time-varying covariate adjustment during follow-up are also key limitations. We lack data on baseline cardiac function parameters, including ECG findings, LVH, or prior history of heart failure or related hospitalizations. Additionally, heart failure-related hospitalization was likely underreported due to non-prespecified data collection.

The yearly mortality rate in AURORA was 12%, thus slightly lower than the yearly mortality of 16% in the European DOPPS registry, suggesting a possible selection bias towards a lower risk in AURORA compared with an unselected HD population [50]. Any findings in the subgroup analyses could be spurious due to multiple testing as these were done upon a review request, data driven, and only presented with some marginally significant interactions.

We conclude that increased levels of hs-cTnI, besides age, are strong and independent risk predictors of future CV events, CV mortality and all-cause mortality in patients with HD, surpassing the traditional and established uraemia-related risk factors. The integration of hs-cTnI into clinical practice has the potential to enhance the management of patients with ESKD on HD, providing a more nuanced understanding of their CV risk and enabling more personalized and effective care.

## Supplementary Material

sfaf047_Supplemental_File

## Data Availability

All data used in this study are available in this article. Because of the sensitive nature of the data collected for this study, requests to access the dataset from qualified researchers trained in human subject confidentiality protocols may be sent to the corresponding author.
