# Supplementary material for: Troponin I is an independent predictor of cardiovascular events and mortality in haemodialysis patients
Source: Clin Kidney J. 2025 Feb 11;18(4):sfaf047. doi: 10.1093/ckj/sfaf047 (PMC12209788; doi:10.1093/ckj/sfaf047)
Supplement: sfaf047_Supplemental_File [file sfaf047_Supplemental_File.docx]

# Appendix 1

## Statistical methods - interaction analysis

Subgroup analyses were performed by including the main effect for each subgroup variable and the interaction effect between the subgroup variable and hs-cTnI. Depending on the model other covariates were included as well as stated above for adjusted models. The same transformations are applied as in the other analyses. The interaction with hs-cTnI was restricted to the linear terms of the components of the restricted cubic spline. For categorical variables, the estimates in each subgroup are estimated from the model using contrasts and applying the appropriate levels for each subgroup (i.e., for the hs-cTnI effect in atrial fibrillation Yes, the contrast is AF = Yes and hs-cTnI Q3 = 32.6 vs AF = Yes and Troponin-i Q1 = 10.1). For continuous variables such as BNP, the interaction is modeled by the linear terms of the components of the restricted cubic splines of both hs-cTnI and BNP. For continuous variables, the contrasts are applied at Q1, median, and Q3 of the interaction variable (i.e., not divided into groups; this is the predicted contrast effect at this BNP value). The results are presented as forest plots and for each subgroup the P-value for interaction (P (int)) is presented.
